# Measurement error in time-series analysis: a simulation study comparing modelled and monitored data

**DOI:** 10.1186/1471-2288-13-136

**Published:** 2013-11-13

**Authors:** Barbara K Butland, Ben Armstrong, Richard W Atkinson, Paul Wilkinson, Mathew R Heal, Ruth M Doherty, Massimo Vieno

**Affiliations:** 1 Division of Population Health Sciences and Education & MRC-PHE Centre for Environment and Health, St George’s, University of London, Cranmer Terrace, Tooting, London SW17 0RE, UK; 2 Department of Social and Environmental Health Research, London School of Hygiene and Tropical Medicine, 15-17 Tavistock Place, London WC1H 9SH, UK; 3 School of Chemistry, University of Edinburgh, Joseph Black Building, West Mains Road, Edinburgh EH9 3JJ, UK; 4 School of GeoSciences, University of Edinburgh, Crew Building, West Mains Road, Edinburgh EH9 3JN, UK; 5 NERC Centre for Ecology & Hydrology, Bush Estate, Nr. Penicuik EH26 0QB, UK

**Keywords:** Measurement error, Epidemiology, Time-series, Mortality, Nitrogen dioxide, Ozone

## Abstract

**Background:**

Assessing health effects from background exposure to air pollution is often hampered by the sparseness of pollution monitoring networks. However, regional atmospheric chemistry-transport models (CTMs) can provide pollution data with national coverage at fine geographical and temporal resolution. We used statistical simulation to compare the impact on epidemiological time-series analysis of additive measurement error in sparse monitor data as opposed to geographically and temporally complete model data.

**Methods:**

Statistical simulations were based on a theoretical area of 4 regions each consisting of twenty-five 5 km × 5 km grid-squares. In the context of a 3-year Poisson regression time-series analysis of the association between mortality and a single pollutant, we compared the error impact of using daily grid-specific model data as opposed to daily regional average monitor data. We investigated how this comparison was affected if we changed the number of grids per region containing a monitor. To inform simulations, estimates (e.g. of pollutant means) were obtained from observed monitor data for 2003–2006 for national network sites across the UK and corresponding model data that were generated by the EMEP-WRF CTM. Average within-site correlations between observed monitor and model data were 0.73 and 0.76 for rural and urban daily maximum 8-hour ozone respectively, and 0.67 and 0.61 for rural and urban log_e_(daily 1-hour maximum NO_2_).

**Results:**

When regional averages were based on 5 or 10 monitors per region, health effect estimates exhibited little bias. However, with only 1 monitor per region, the regression coefficient in our time-series analysis was attenuated by an estimated 6% for urban background ozone, 13% for rural ozone, 29% for urban background log_e_(NO_2_) and 38% for rural log_e_(NO_2_). For grid-specific model data the corresponding figures were 19%, 22%, 54% and 44% respectively, i.e. similar for rural log_e_(NO_2_) but more marked for urban log_e_(NO_2_).

**Conclusion:**

Even if correlations between model and monitor data appear reasonably strong, additive classical measurement error in model data may lead to appreciable bias in health effect estimates. As process-based air pollution models become more widely used in epidemiological time-series analysis, assessments of error impact that include statistical simulation may be useful.

## Background

Bias in estimation due to measurement error has received much attention in medical research including epidemiology. In its simplest form i.e. pure additive classical measurement error, the relationship between the observed variable or surrogate measure *Z* and the “true” variable X^*^ can be expressed as:

(1.1) $$Z=X^{*}+\gamma$$

$$\gamma ∼Ν\left(0,\sigma_{d}^{2}\right),\;\mathrm{cov}\left(X^{*},\gamma \right)=0,\;E\left(Z\right)=E\left(X^{*}\right)$$

It is well documented that replacing X^*^ by *Z* as the explanatory variable in a simple linear regression analysis leads to attenuation in the estimation of both the Pearson correlation coefficient and the gradient of the regression line with the extent of the attenuation depending on the reliability ratio *ρ*_ZX*_ where *ρ*_*ZX* *_ = *var*(*X*^∗^)/*var*(*Z*) [1]. Similarly in simple Poisson regression pure additive classical error in the explanatory variable leads to attenuation in the estimation of the relative risk [2].

However, not all measurement error is classical. Reeves et al. [3] considered the impact of measurement error in a situation where individual radon exposure was measured with additive classical error but where subjects with missing radon data were assigned an area average. If the variability of “true” individual radon exposure is the same within each area and the area averages are exact (i.e. measured without error) their use as surrogate measures introduces pure additive Berkson error. This type of measurement error has no biasing effect on the regression coefficient in simple linear regression [4] and little if any such effect on the regression coefficient in simple Poisson regression [2,5]. However if the averages are not exact they introduce a combination of Berkson error and classical error and the presence of additive classical error biases the gradient estimate or relative risk estimate towards the null.

The consequences of using an area average as a surrogate explanatory variable has been investigated in simulations by Lee et al. [6]. Based on a time-series analysis of daily mortality counts and average daily air pollution (average of readings from available monitors in the study region), they found that increasing the probability of siting monitors in high pollution areas led to attenuation in health effect estimates and poor coverage intervals. They also found that within a separate scenario of high classical instrument error (assumed to be additive on a log scale) and low spatial variation, reducing the total number of monitors in the study region from 30 to 5 enhanced any attenuation in the health effect estimate.

As indicated above, in some circumstances measurement error is proportional (i.e. additive on a log scale) and the relationship of interest is with the untransformed explanatory variable. In the context of using over-dispersed Poisson regression to investigate the effects of air pollution on daily emergency department visits, a recent simulation study by Goldman et al. [5] concluded that while pure proportional classical error in the daily air pollution data led to an attenuated estimate of relative risk, pure proportional Berkson error in the pollution data actually led to an inflated estimate of relative risk, i.e. bias away from the null. This is in line with findings for logistic regression from the simulation study of Steenland et al. [7] which suggested that if the Berkson error variance increases as values of the surrogate measure increase, bias in the regression coefficient away from the null may result.

For statistical models containing more than one explanatory variable, the effect of measurement error depends not only on the error type (i.e. Berkson, classical, proportional, additive) but also on the correlation between the explanatory variables, which explanatory variables are causal and which are measured with error. In Poisson regression with two explanatory variables, one causal and measured with pure additive classical error and the other non-causal and measured without error, Fung et al. [8] demonstrated through simulation that the estimated relative risk of the causal variable will be attenuated and that if the correlation between the two explanatory variables is high (i.e. multicollinearity) the predictive effect of the causal variable may be transferred to the non-causal variable.

In air pollution epidemiology short-term associations between outdoor air pollution and health are assessed using an ecological time-series design. Many such studies have been published [9] and inform public health policy [10]. These studies correlate daily counts of health events in a specific location (usually a city) with daily pollution concentrations derived from static monitoring sites. Regional air pollution chemistry-transport models (CTMs) that are capable of simulating hourly and daily concentrations of a wide range of pollutants at fine-scale resolutions (i.e. ≤ 10 km) have recently been developed. These provide new opportunities to investigate pollution metrics (e.g. individual particulate matter components or source-resolved pollutant metrics) which either cannot be currently measured or can be measured only at a limited number of locations due to their measurement complexity and/or a sparse monitoring network. In this paper we compare, using statistical simulation methods, the performance of geographically resolved model data at 5 km × 5 km resolution with area-wide average concentrations derived from a number of air pollution monitors.

We compare performance in terms of additive and proportional measurement error and its effect on a time-series analysis of the relationship between daily ambient background pollution levels and daily counts of a health event (using all-cause mortality as an example) at the small area level, i.e. the 5 km × 5 km grid. The analysis is conducted using Poisson regression. Measures from background air pollution monitors may be available for some 5 km × 5 km grids but not for others. Our primary aim is therefore to demonstrate how simulation techniques can be employed to investigate when and if it might be better to use data from a CTM rather than as often happens in practice, aggregating over grids and using average pollution values based on monitor data. Our simulations are based on a theoretical study area divided into 4 regions each consisting of twenty-five 5 km × 5 km grids and within this construct we consider the effect of varying the number of grids per region containing a monitor. The parameter estimates used in our simulations are taken from observed monitor versus model comparisons.

For the purposes of this investigation we assume that it is the association of ambient pollution with mortality at the small-area level that is important (because of the link to regulation, [11]) rather than exposure at the level of the individual and leave consideration of disparities between background monitoring networks and personal exposure to others [4,11,12]. There is also a literature on impacts of measurement error in air pollution for study designs other than time-series [13,14].

## Methods

### Simulating a “true” time-series

Simulations were performed using DRAWNORM in STATA 10 [15] and relate to a theoretical square study area measuring 50 km North by 50 km East which can be divided into 4 regions each consisting of twenty-five 5 km × 5 km grid-squares. We assume that:

● For a given pollutant its “true” background concentration (i.e. devoid of bias or measurement error) in grid-square *i* on day *t* of a 3-year time-series is:

$$x_{i,t}^{*}\left(i=1,\ldots ,100;t=1,\ldots ,1095\right)$$

● For each grid *i*, the “true” 3-year time-series, represented by the vector $X_{i}^{*}$ , exhibits no trend or seasonal variation.

● 

$$X_{i}^{*}=\;\left[\begin{array}{l} \;x_{i,1}^{*}\\ \;\cdot \\ \;\cdot \\ \;\cdot \\ x_{i,1095}^{*}\; \end{array}\right]$$

● Grid-specific means *μ*_*i*_ (*i* = 1, …, 100) are Normally distributed around an overall mean *μ* with variance $\sigma_{b}^{2}$.

$$\mu_{i}=\mu +e_{i},\;e_{i}∼N\left(0,\sigma_{b}^{2}\right)$$

● Each row of the 1095 × 100 time-series matrix, $X^{*}=\left[X_{1}^{*},\ldots ,X_{100}^{*}\right]$, consists of a sample drawn from a Multivariate Normal distribution, *MVN*(U, **Ω**), with grid-specific means, *μ*_*i* (*i* = 1,…,100)_, common within-grid variance $\sigma_{w}^{2}$, between-grid covariances *σ*_*i*,*k* (*i* = 1,…,100;*k* = 1,…,100)_ and between-grid correlation coefficients *ρ*_*i*,*k* (*i* = 1,…,100;*k* = 1,…,100)_, such that:

$$U=\;\left[\begin{array}{l} \mu_{1}\\ \;\cdot \\ \;\cdot \\ \;\cdot \\ \mu_{100} \end{array}\right]\;,\;Ω=\left[\begin{array}{ccccc} \sigma_{w}^{2} & \cdot & \cdot & \cdot & \sigma_{100,1}\\ \cdot & \cdot & \cdot & \cdot & \cdot \\ \cdot & \cdot & \cdot & \cdot & \cdot \\ \cdot & \cdot & \cdot & \cdot & \cdot \\ \sigma_{1,100} & \cdot & \cdot & \cdot & \sigma_{w}^{2} \end{array}\right]$$

● For each grid *i* the number of deaths on day *t*, *y*_*i,t*_, is sampled from a Poisson distribution with mean dependent on the “true” background concentration of the pollutant in that grid on that day, according to the following formula:

$$E\left(Y_{i,t}\right)=\varphi_{i,t}=\alpha \times \exp\left(\beta \times x_{i,t}^{*}\right)$$

$$Y_{i,t}\sim \mathrm{Poisson}\;\left(\varphi_{i,t}\right)$$

We consider two pollutant metrics, daily maximum 8-hour ozone and log_e_(daily 1-hour maximum NO_2_). NO_2_ concentrations are log transformed to take account of a positively skewed distribution.

For ozone we set *α* = 0.32 (i.e. mean daily deaths with 0 *μ*g/m^3^ ozone = 0.32) and *β* = 0.0003992 (*i. e. e*^*β* × 10^ = 1.0040). While values assigned to *α* and *β* are somewhat arbitrary, a 0.4% increase in mortality per 10 *μ*g/m^3^ increase in ozone,  (*i. e. β* = 0.0003992), is the size of effect that might be observed in a real epidemiological study [9]. For log_e_(NO_2_), to aid the comparison of findings in tables, we set *α* = 0.32 (i.e. mean daily deaths with 1 *μ*g/m^3^ NO_2_ = 0.32) and *β* = 0.0418845 (*i. e*. 1.10^*β*^ = 1.0040 indicating a 0.4% increase in mortality per 10% increase in NO_2_).

### Simulating observed monitor data

Pollution concentrations obtained from monitors will include measurement error due to instrument imprecision and monitor location. Given the small size of grids (i.e. 5 km × 5 km) and that instrument error for an unbiased monitor is generally considered to be classical [16], for each grid *i* we simulate a 3-year time-series of monitor data, *X*_*i*_, by adding classical measurement error to our “true” time-series $X_{i}^{*}$ as follows:

$$X_{i}=X_{i}^{*}+E_{i}$$

where for each element *ϵ*_*i*,*t*_ of the error vector *E*_*i*_

$$\epsilon_{i,t}\sim N\left(0,\sigma_{\mathrm{err}}^{2}\right)$$

such that,

$$E\left(X_{i}\right)=E\left(X_{i}^{*}\right)=\mu_{i}$$

### Simulating model data

For each grid *i* we simulate a 3-year time-series of model data*, Z*_*i*_, from $X_{i}^{*}$. However in contrast to the above we allow for a grid-specific bias (i.e. $E\left(X_{i}^{*}\right)=\mu_{i},\;E\left(Z_{i}\right)=\mu_{i}+c_{i}$, where *μ*_*i*_ and *c*_*i*_ are grid-specific constants) and for the presence of Berkson-like error as well as classical-like error (i.e. we allow for the possibility that, $\mathrm{cov}\left(X_{i}^{*},Z_{i}\right)\neq \mathrm{var}\left(X_{i}^{*}\right)$). We do this by using the approach of Reeves et al. [3]. This approach exploits the fact that if we express *Z*_*i*_ as a linear function of $X_{i}^{*}$ then using standard theory as outlined in Cox and Hinkley [17]:

(1.2) $$Z_{i}=c_{i}+\mu_{i}+\left\{\frac{\mathrm{cov}\left(X_{i}^{*},Z_{i}\right)}{\mathrm{var}\left(X_{i}^{*}\right)}\right\}\left(X_{i}^{*}-\mu_{i}\right)+\Delta_{i}$$

where,

$$\Delta_{i}=\left[\begin{array}{c} \delta_{i,1}\\ \begin{array}{c} .\\ .\\ . \end{array}\\ \delta_{i,1095} \end{array}\right]$$

$$\delta_{i,t}\sim N\left(0,\sigma_{i,z.x^{*}}^{2}\right)$$

, $\mathrm{cov}\left(\Delta_{i},X_{i}^{*}\right)=0\;$ and $\sigma_{i,z.x^{*}}^{2}=\mathrm{var}\left(Z_{i}\right)-{\left\{\frac{\mathrm{cov}\left(X_{i}^{*},Z_{i}\right)}{\mathrm{var}\left(X_{i}^{*}\right)}\right\}}^{2}\mathrm{var}\left(X_{i}^{*}\right)$.

If there is no Berkson-like error (i.e. $\mathrm{cov}\left(X_{i}^{*},Z_{i}\right)=\mathrm{var}\left(X_{i}^{*}\right))$ then with the exception of the grid-specific bias term (*c*_*i*_) formula 1.2 reduces to a classical error model.

In populating 1.2, we assume that model data are uncorrelated with instrument and location error (i.e. *cov*(Ε_*i*_, *Z*_*i*_) = 0). From this it follows that $\mathrm{cov}\left(X_{i},Z_{i}\right)=\mathrm{cov}\left(X_{i}^{*}+{Ε}_{i},Z_{i}\right)=\mathrm{cov}\left(X_{i}^{*},Z_{i}\right)+\mathrm{cov}\left({Ε}_{i},Z_{i}\right)=\mathrm{cov}\left(X_{i}^{*},Z_{i}\right)$. In addition, provided our focus is on the effects of additive measurement error, (not the case for proportional measurement error), and our time-series analysis adjusts for grid, we can simplify calculations by setting the grid-specific constant terms *c*_*i*_ = *c* for all *i* = 1, …, 100.

For the purposes of our simulations involving proportional error we ignore any dependence between $E\left(Z_{i}\right)-E\left(X_{i}^{*}\right)$ and $E\left(X_{i}^{*}\right)$ and assume that:

(1.2a) $$c_{i}=c+\epsilon_{c},\;\epsilon_{c}\sim N\left(0,\sigma_{\mathrm{diff}}^{2}\right)$$

### Simulating regional averages

We simulate the use of regional averages in situations where pollution monitor coverage is less than 1 monitor per 5 km × 5 km grid by first sampling a sub-set of *l* grid-squares (*R*_*jl*_(*j* = 1, …, 4)) from each of the 4 regional sets of 25 grids-squares (*R*_*j*_(*j* = 1, …, 4)) such that  *R*_*jl*_ ⊂ *R*_*j*_. Next we replace each 3-year time-series,  *X*_*i*_(*i ∊ R*_*j*_*)* with a 3-year time-series of averages *W*_*j*_ based on the formula:

$$W_{j}=\frac{1}{l}\sum_{i\in R_{\mathrm{jl}}}X_{i\;}\;j=1,\ldots ,4$$

Simulated regional average time-series are produced in this way for *l* = 5, *l* = 10, *l* = 15, *l* = 20, *l* = 25.

We also consider the single monitor scenario i.e. *l* = 1.

### Comparison of observed monitor and CTM data

Realistic estimates for the above as yet unset parameters (e.g. $\;\sigma_{b}^{2},\mathrm{var}\left(Z_{i}\right)$) were obtained by reference to observed monitor and chemistry-transport model (CTM) data. The monitor data came from the UK’s Automatic Urban and Rural Network (AURN) and were obtained via the UK national air information resource [18].

The modelled data used were daily outputs from the EMEP-WRF v3.7 grid-based (Eulerian) 3-D CTM which provides a detailed simulation of the evolving physical and chemical state of the atmosphere over the UK. The underlying CTM is the EMEP Unified Model [19] which has been modified to enable application at 5 km horizontal spatial resolution over the British Isles [20]. A nested approach is used whereby EMEP simulations of atmospheric composition across a coarser European domain are used to drive fine-scale EMEP-WRF simulations of air quality at 5 km horizontal resolution across the UK. The EMEP and EMEP-WRF models have been extensively validated and used for numerous policy applications [21,22].

Daily concentrations of monitored ozone  (*μ*g/m^3^) and their corresponding EMEP-WRF CTM estimates, covering a total of at least 364 days over the period 2003–2006, were obtained for 35 urban background and 21 rural monitoring sites across England, Wales, Scotland and Northern Ireland. Similarly paired daily concentrations of NO_2_ (*μ*g/m^3^), again covering at least 364 days over the period 2003–2006, were obtained for 43 urban background and 14 rural monitoring sites across, England, Wales, Scotland and Northern Ireland. Ozone concentrations were daily maximum running 8-hour mean and NO_2_ concentrations were log_e_-transformed (daily 1-hour maximum). Summary statistics comparing monitor and CTM data for rural and urban sites are presented in Table 1.

**Table 1:** Comparison of observed model and chemistry-transport model (CTM) data

**Variables**	**Site type (No. of sites)**	**Total No. of days across sites**	**Source of data**	**Average (SD) of site specific means**	**Average (SD) of within-site standard deviations**	**Average within-site covariance between monitor and CTM data**	**Average within-site correlation between monitor and CTM data**	**Average within-site correlation with differences (CTM-monitor)**	**Average (SD) of site-specific mean difference [CTM-monitor]**
Daily maximum running 8-hour mean^§^ O_3_	Rural (N=21)	26,995	Monitor	72.17 (5.10)	21.65 (3.88)	334.927	0.730	−0.424	4.740 (4.375)
			CTM	76.91 (1.81)	20.50 (2.98)			0.304	
	Urban (N=35)	40,938	Monitor	61.73 (7.38)	25.28 (2.81)	455.779	0.757	−0.442	10.250 (5.166)
			CTM	71.98 (3.93)	23.41 (2.92)			0.246	
log_e_(Daily maximum 1-hour^¶^ NO_2_)	Rural (N=14)	16,080	Monitor	2.696 (0.495)	0.705 (0.110)	0.422	0.667	−0.194	−0.236 (0.210)
			CTM	2.460 (0.588)	0.866 (0.146)			0.595	
	Urban (N=43)	51,596	Monitor	3.857 (0.309)	0.473 (0.094)	0.195	0.612	−0.158	−0.538 (0.268)
			CTM	3.319 (0.438)	0.645 (0.138)			0.674	

^¶^ Values on 35 days set to missing as daily monitor maximum 1-hour NO_2_=0. ^§^The 8-hour rolling mean ozone concentration assigned to hour *h* is the average of hourly concentrations at *h*-7, *h*-6, *h*-5, *h*-4, *h*-3, *h*-2, *h*-1, and *h*. In calculating pollution metrics we employ the 75% rule such that a valid 8-hour mean must be based on at least 6 values, a daily maximum 8-hour mean must be based on at least 18 valid 8-hour means and a daily maximum 1-hour concentration on at least 18 hourly concentrations.

**Figure 1:**
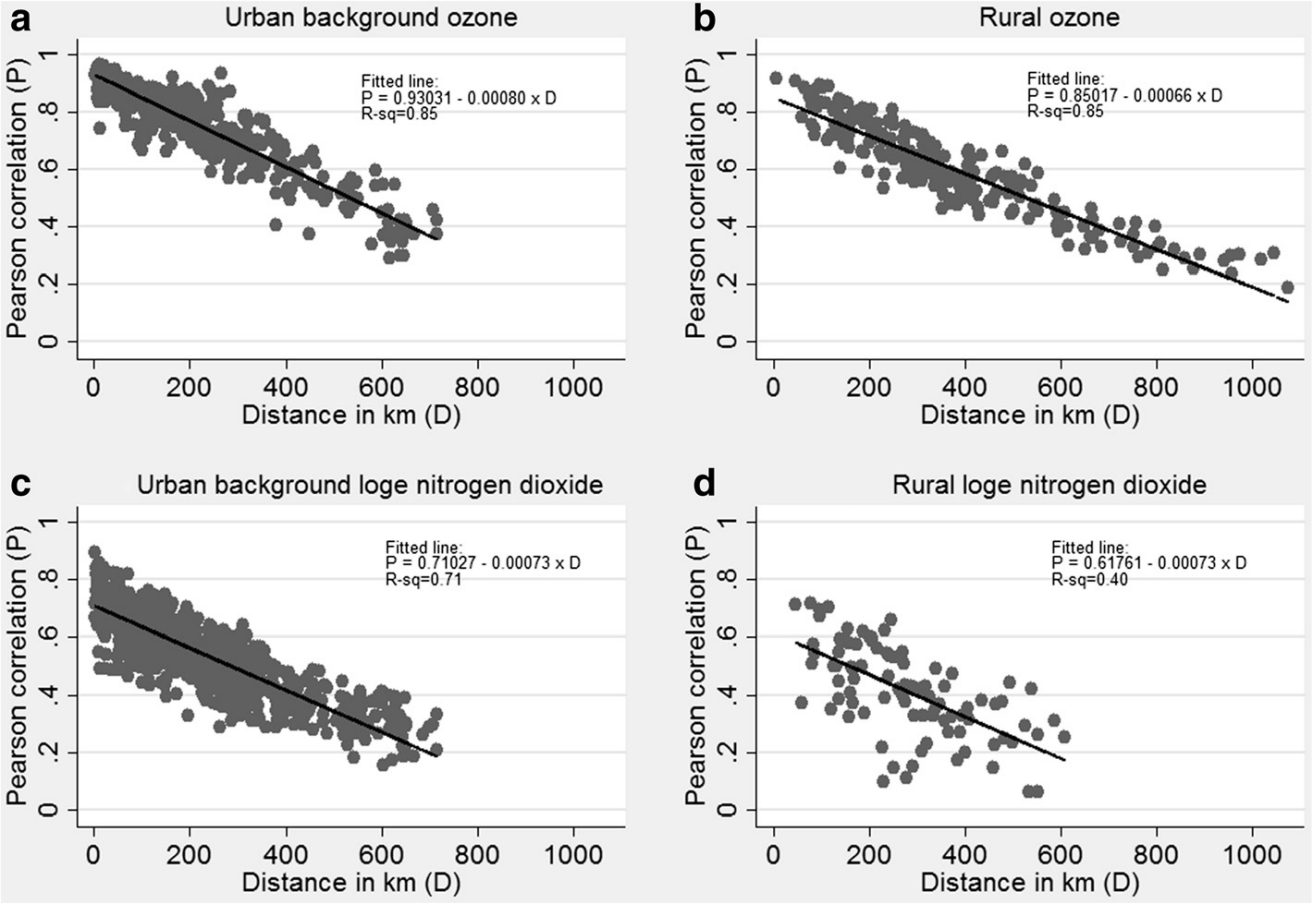
**Simple linear regression analysis of Pearson correlation by distance.** The figure presents results for **(a)** urban background ozone, **(b)** rural ozone, **(c)** urban background log_e_(NO_2_) and **(d)** rural log_e_(NO_2_). Each point on graphs represents the Pearson correlation (P) between daily standardised pollution concentrations measured at two distinct monitoring sites, plotted against the distance in km (D) between those sites. R-sq: estimate of the proportion of variance in Pearson correlation (P) explained by the fitted linear relationship with distance in km (D).

The distance between each pair of monitoring sites of the same type was calculated. Then having first standardised monitored pollution concentrations within site by subtracting the site mean and dividing by the site standard deviation, Pearson correlations across time between site pairs were calculated for rural ozone, urban background ozone, rural log_e_(NO_2_) and urban background log_e_(NO_2_) and plotted against distance (Figure 1). Correlations based on <364 paired observations were set to missing. The relationships between Pearson correlation and distance were then investigated using simple linear regression.

### Parameter estimates

To simulate “true” urban background ozone concentrations for our theoretical study area we set *μ* = 61.73 and $\sigma_{b}^{2}=7.38^{2}$ (Table 1), and constructed a correlation matrix ***ρ*** (100,100) using the regression equation based on Pearson correlation as a function of distance between monitors (Figure 1(a)):

$$E\left[P\right]=0.93031-0.00080\times D$$

Each off-diagonal element of ***ρ*** was calculated by setting *D* equal to an estimate of the average distance in km *between* any two points, one in each of the two 5 km × 5 km grid-squares being compared (using simulation: $D\approx d+2.13\times \left(\frac{1}{d}\right)$ where *d* is the straight line distance between the centre points of the two grid-squares). The diagonal elements were calculated by setting *D* equal to an estimate of the average distance between any two points *within* a 5 km × 5 km grid-square (using simulation: *D* ≈ 2.6 ). The variance/covariance matrix **Ω**(100, 100) was then obtained by multiplying each element of ***ρ*** by the average observed within-site variance (i.e. 25.28^2^ in Table 1). This produced a symmetrical matrix with diagonal elements equal to 24.35^2^, the estimated average “true” within-site variance having removed any variation due to instrument error and monitor-site location error (i.e. $\;\sigma_{\mathrm{err}}^{2})$.

For simulating observed monitor data we set  *σ*_*err*_ = 6.77 (see Additional file 1) and for simulating model data within each grid *i* we set: $\mathrm{cov}\left(X_{i}^{*},Z_{i}\right)=455.78,\mathrm{var}\left(X_{i}^{*}\right)=24.35^{2},$*var*(*Z*_*i*_) = 23.41^2^, and *c* = 10.25 (see Table 1). Parameter estimates for rural ozone, urban background log_e_(NO_2_) and rural log_e_(NO_2_) were obtained in the same fashion.

### Proportional measurement error

For NO_2_ we have assumed that measurement error is additive on a log scale and that the relationship of interest is with log_e_(NO_2_). If, however, the relationship of interest is with NO_2_ (untransformed) then measurement error in the explanatory variable is proportional rather than additive. In order to simulate monitor NO_2_ data with proportional error, we first simulate log_e_(NO_2_) as before but then back-transform (i.e. NO_2_ = exp(log_e_(NO_2_)) prior to calculating regional averages. For model data, we first simulate log_e_(NO_2_) as in Equation (1.2) but instead of setting the  *c*_*i*_ = *c*, we use Equation (1.2a) and set *σ*_*diff*_ = 0.268 for urban background log_e_(NO_2_) and *σ*_*diff*_ = 0.210 for rural log_e_(NO_2_) (see Table 1). The data are then back-transformed. With NO_2_ rather than log_e_(NO_2_) as the explanatory variable in our epidemiological time-series analysis we set: *α* = 0.32 and *β* = 0.0003992 (*i. e. e*^*β* × 10^ = 1.0040 indicating a 0.4% increase in mortality per 10 *μ*g/m^3^ increase in NO_2_).

### Statistical analysis of simulated time series

For each of the 7 time-series scenarios considered in each of Tables 2, 3 and 4, 1000 simulated data sets were produced and each analysed separately using Poisson regression with grid as a fixed effect. As a result, 1000 separate estimates of both the health effect ($\hat{\beta }$) and its standard error, $\mathrm{SE}\left(\hat{\beta }\right)$, were obtained. Statistics presented in Tables 2, 3 and 4 include estimate averages and estimates of the coverage probability and power. An estimate of coverage probability records the percentage of simulations where the 95% confidence interval contains the “true” value of *β* and an estimate of power records the percentage of simulations that would have detected the health effect estimate as statistically significant at the 5% significance level.

**Table 2:** Summarising the analysis of 1000 simulated data sets: urban background pollution concentrations with additive error

**Description of simulated data**	**No. of grids per region containing a monitor**	**Ozone *β * × 10 = 0.00399**		**log**_**e**_**(Nitrogen Dioxide) *β * = 0.0419**	
		$\hat{\beta }\times 10$	**Coverage probability;**	$\hat{\beta }$	**Coverage probability;**
		$\mathrm{SE}\left(\hat{\beta }\right)\times 10$	**Power**	$\mathrm{SE}\left(\hat{\beta }\right)$	**Power**
***Monitor data:*** regional average used for each 5 km × 5km grid within region (instrument and monitor-site location error included)	1	0.00375 (0.00209)	94%; 43%	0.0297 (0.0104)	78%; 81%
	2	0.00388 (0.00214)	95%; 44%	0.0348 (0.0113)	91%; 86%
	3	0.00393 (0.00215)	95%; 45%	0.0369 (0.0117)	93%; 89%
	5	0.00396 (0.00216)	96%; 45%	0.0387 (0.0120)	94%; 90%
	10	0.00400 (0.00217)	95%; 45%	0.0403 (0.0122)	95%; 90%
***“true” data:*** grid-specific monitor data (no instrument or monitor-site location error)	25	**0.00394 (0.00217)**	**95%; 45%**	**0.0417 (0.0124)**	**95%; 93%**
***Model data:*** grid-specific model data	-	0.00325 (0.00226)	94%; 29%	0.0193 (0.0076)	15%; 72%

The table presents estimated regression coefficients $\left(\hat{\beta }\right)$, standard errors $\left(\mathrm{SE}\left(\hat{\beta }\right)\right)$, coverage probabilities and power, each based on the analysis of 1000 sets of simulated time-series data. The “true” value of the regression coefficient *β* for ozone (i.e. *β* × 10 = 0.00399) equates to a 0.4% increase in mortality per 10 *μ*g/m^3^ increase in ozone and the “true” value of the regression coefficient for log_e_(NO_2_) (i.e.  *β* = 0.0419) equates to a 0.4% increase in mortality per 10% increase in NO_2_.

**Table 3:** Summarising the analysis of 1000 simulated data sets: rural pollution concentrations with additive error

**Description of simulated data**	**No. of grids per region containing a monitor**	**Ozone** ***β*** **× 10 = 0.00399**		**log**_**e**_**(Nitrogen Dioxide)** ***β*** **= 0.0419**	
		$\hat{\beta }\times 10$	**Coverage probability;**	$\hat{\beta }$	**Coverage probability;**
		$\mathrm{SE}\left(\hat{\beta }\right)\times 10$	**Power**	$\mathrm{SE}\left(\hat{\beta }\right)$	**Power**
***Monitor data:*** regional average used for each 5 km × 5km grid within region (instrument and monitor-site location error included)	1	0.00346 (0.00244)	95%; 30%	0.0258 (0.0072)	39%; 96%
	2	0.00371 (0.00254)	95%; 31%	0.0319 (0.0080)	76%; 98%
	3	0.00381 (0.00258)	95%; 31%	0.0347 (0.0083)	86%; 98%
	5	0.00389 (0.00261)	95%; 32%	0.0372 (0.0087)	93%; 99%
	10	0.00397 (0.00263)	96%; 31%	0.0395 (0.0089)	95%; 99%
***“true” data:*** grid-specific monitor data (no instrument or monitor-site location error)	25	**0.00392 (0.00264)**	**95%; 33%**	**0.0418 (0.0091)**	**94%; 100%**
***Model data:*** grid-specific model data	-	0.00310 (0.00257)	94%; 22%	0.0233 (0.0058)	11%; 99%

The table presents estimated regression coefficients $\left(\hat{\beta }\right)$, standard errors $\left(\mathrm{SE}\left(\hat{\beta }\right)\right)$, coverage probabilities and power, each based on the analysis of 1000 sets of simulated time-series data. The “true” value of the regression coefficient *β* for ozone (i.e. *β* × 10 = 0.00399) equates to a 0.4% increase in mortality per 10 *μ*g/m^3^ increase in ozone and the “true” value of the regression coefficient for log_e_(NO_2_) (i.e.  *β* = 0.0419) equates to a 0.4% increase in mortality per 10% increase in NO_2_.

**Table 4:** Summarising the analysis of 1000 simulated data sets: nitrogen dioxide concentrations with proportional error

**Description of simulated data**	**No. of grids per region containing a monitor**	**Urban background Nitrogen Dioxide** ***β*** **× 10 = 0.00399**		**Rural background Nitrogen Dioxide *β * × 10 = 0.00399**	
		$\hat{\beta }\times 10$	**Coverage probability;**	$\hat{\beta }\times 10$	**Coverage probability;**
		$\mathrm{SE}\left(\hat{\beta }\right)\times 10$	**Power**	$\mathrm{SE}\left(\hat{\beta }\right)\times 10$	**Power**
***Monitor data:*** regional average used for each 5 km × 5km grid within region (instrument and monitor-site location error included)	1	0.00262 (0.00191)	86%; 28%	0.00201 (0.00319)	86%; 10%
	2	0.00313 (0.00207)	91%; 32%	0.00252 (0.00350)	92%; 11%
	3	0.00333 (0.00213)	93%; 35%	0.00279 (0.00365)	93%; 12%
	5	0.00353 (0.00219)	94%; 37%	0.00306 (0.00381)	93%; 13%
	10	0.00372 (0.00224)	95%; 38%	0.00335 (0.00396)	94%; 14%
***“true” data:*** grid-specific monitor data (no instrument or monitor-site location error)	25	**0.00394 (0.00225)**	**95%; 43%**	**0.00393 (0.00403)**	**95%; 17%**
***Model data:*** grid-specific model data	-	0.00231 (0.00182)	85%; 25%	0.00185 (0.00222)	83%; 14%

The table presents estimated regression coefficients $\left(\hat{\beta }\right)$, standard errors $\left(\mathrm{SE}\left(\hat{\beta }\right)\right)$, coverage probabilities and power, each based on the analysis of 1000 sets of simulated time-series data. The “true” value of the regression coefficient *β* for NO_2_ (i.e. *β* × 10 = 0.00399) equates to a 0.4% increase in mortality per 10 μg/m^3^ increase in NO_2_.

Finally using established theory (See Additional file 2) we obtained predictions of the attenuation in *β* that we might expect from using CTM data or data from a single monitor per region. These predictions were then compared to the corresponding results obtained from our simulations.

### Error decomposition

In order to aid interpretation of our simulation results for the CTM data, we decomposed the grid-specific error variance $\mathrm{var}\left(Z_{i}-X_{i}^{*}\right)$ into two components, a classical-like component (CC), and a Berkson-like component (BC) as follows:

$$\begin{array}{l} \mathrm{var}\left(Z_{i}-X_{i}^{*}\right)=\mathrm{cov}\left(Z_{i}-X_{i}^{*},Z_{i}\right)+\left\{-\mathrm{cov}\left(Z_{i}-X_{i}^{*},X_{i}^{*}\right)\right\}\\ \;=\mathrm{CC}+\mathrm{BC} \end{array}$$

where

$$\mathrm{CC}=\mathrm{cov}\left(Z_{i}-X_{i}^{*},Z_{i}\right)=\left\{\mathrm{var}\left(Z_{i}\right)-\mathrm{cov}\left(Z_{i},X_{i}^{*}\right)\right\}$$

and

$$\mathrm{BC}=\left\{-\mathrm{cov}\left(Z_{i}-X_{i}^{*},X_{i}^{*}\right)\right\}=\left\{\mathrm{var}\left(X_{i}^{*}\right)-\mathrm{cov}\left(Z_{i},X_{i}^{*}\right)\right\}$$

Estimates of CC and BC were then obtained using the observed data (See Additional file 3 for further details and calculations).

## Results

Comparing “true” values of the regression coefficient, *β,* (e.g.  *β* × 10 = 0.00399 for urban background ozone) with those based on simulated data, $\left(\hat{\beta }\right)$, Tables 2 and 3 suggest that the use of regional average monitor data as a surrogate for grid-specific “true” ambient concentrations has limited impact on health effect estimates unless the number of monitors per 25 km × 25 km grid-square falls below 3 (or possibly 5 in the case of rural log_e_(NO_2_)). The monitoring scenario which produced the largest bias in the health effect for all four pollutants was that of a single monitor per 25 km × 25 km grid-square. The regression coefficient was attenuated by an estimated 6% for urban ozone, 13% for rural ozone, 29% for urban log_e_(NO_2_) and 38% for rural log_e_(NO_2_). By contrast when we used grid-specific model data, the regression coefficient was attenuated by an estimated 19% for urban ozone, 22% for rural ozone, 54% for urban log_e_(NO_2_) and 44% for rural log_e_(NO_2_). Thus, although for rural log_e_(NO_2_) results were similar to those of the 1 monitor per region scenario, for urban and rural ozone, urban log_e_(NO_2_) and for less sparse monitoring networks the use of model rather than monitor data appeared to produce a more marked level of bias in the health effect estimate. Comparison of the “true” values of the regression coefficient with those based on simulated “true” data (Tables 2 and 3) suggests that our findings are not simply due to an inadequate number of simulations.

Of particular note are the small coverage probabilities for log_e_(NO_2_), especially when using the grid-specific model data, but also evident when using measured rural data from a single monitor within each 25 km × 25 km grid. These suggest that not only is there marked attenuation in the health effect estimate but that bias extends to the standard errors, such that few simulations produced a 95% confidence interval containing the “true” value of *β* (only 15% for urban background modelled log_e_(NO_2_) and 11% for rural modelled log_e_(NO_2_) (Tables 2 and 3). As expected statistical power for log_e_(NO_2_) is consistently higher than for ozone as the magnitude of the “true” effect to be detected is larger (i.e. a 0.4% increase in mortality per 10% increase in NO_2_ versus a 0.4% increase in mortality per 10 *μ*g/m^3^ in ozone). Nevertheless, the use of grid-specific model data for urban and rural ozone and the use of either model or 1 monitor per region data for urban log_e_(NO_2_) appears to have a slightly adverse effect on power.

Table 4 presents results for NO_2_ assuming proportional measurement error (i.e. additive on a log scale) but where the relationship of interest is with the untransformed variable. Overall, compared to log_e_(NO_2_), power-loss due to measurement error was similar but coverage probabilities, particularly for model data, improved. Model data and the single monitor scenario registered the largest attenuation in the regression coefficient, but there was noticeable attenuation even when using regional averages based on 5 monitors per 25 km × 25 km region.

### Predictions from theory

For model data and for the 1 monitor scenario, established theory (see Additional file 2) allows us to predict the effects of additive measurement error on the health effect estimate. Table 5 illustrates that estimates of attenuation in *β* obtained by simulation are not that dissimilar from those obtained using standard theory in this simple case.

**Table 5:** Estimated attenuation in the health effect estimate: comparing simulation and theory

**Data description**		**Ozone *β * × 10 = 0.00399**		**log**_**e**_**(Nitrogen Dioxide) *β * = 0.0419**	
		**Simulation**	**Theory**	**Simulation**	**Theory**
		$\hat{\beta }\times 10$	$\hat{\beta }\times 10$	$\hat{\beta }$	$\hat{\beta }$
***Monitor data:*** measurements from a single monitor used for each 5 km × 5km grid within region (instrument and monitor-site location error included)	Urban background	0.00375	0.00367	0.0297	0.0293
	Rural	0.00346	0.00336	0.0258	0.0255
***Model data:*** grid-specific model data	Urban background	0.00325	0.00332	0.0193	0.0196
	Rural	0.00310	0.00318	0.0233	0.0236

For model data we base our predictions on the average observed within-site covariance rather than the average observed within-site correlation.

## Discussion

In the context of a time-series analysis of the association between daily concentration of air pollution and mortality, our study used simulation as a technique to contrast the effects on the estimation of that association of using grid-specific pollution data derived from a 3-D chemistry-transport model as opposed to regional average air pollution concentrations derived from monitors. Pollution concentrations were simulated both with (i.e. monitor data), and without (i.e. “true” data) classical “instrument and monitor-location” error. The “true” data were then used in the statistical simulation of model data with the inclusion of both classical and Berkson-like error. The parameter estimates driving our simulations were based on both monitor and CTM daily maximum 8-hour mean ozone data for 35 urban background and 21 rural monitoring sites across the UK and on both monitor and CTM log_e_(daily maximum 1-hour NO_2_) data for 43 urban background and 14 rural monitoring sites across the UK. Within-grid correlations between observed monitor and CTM data were relatively strong with average correlation coefficients of 0.73 for rural ozone, 0.76 for urban ozone, 0.67 for rural log_e_(NO_2_) and 0.61 for urban log_e_(NO_2_). The lower correlations for log_e_(NO_2_) were likely a consequence of the shorter averaging time of the NO_2_ metric (i.e. 1-hour rather than 8-hour for ozone).

For both pollutants (i.e. ozone and log_e_(NO_2_)), the use of a single monitor to provide estimated pollution concentrations for every 5 km × 5 km grid within a 25 km × 25 km region produced attenuated health effect estimates. This attenuation was less marked for the more spatially homogeneous long-lived pollutant ozone, for which the short distance correlations in Figure 1 were strong, than for the short-lived pollutant log_e_(NO_2_) for which the short distance correlations were considerably weaker. However for other scenarios, particularly those based on 5 or 10 monitors, the use of regional averages with additive rather than proportional error had little effect on health effect estimates. This concurs with the simulation findings of Sheppard et al. [12] who reported a “small but noticeable” attenuation in the heath effect estimate when ambient area exposure to PM_2.5_ was based on a single pollution monitor, but little if any attenuation when area exposure was based on the average across 3 or 10 monitors.

Goldman et al. [16] recognized that a large proportion of the measurement error introduced by the use of average monitor concentrations is due to spatial variation and suggests that such error is predominantly Berkson, which, while reducing statistical power, will not on its own lead to bias in health effect estimates. However as classical error is introduced, occurring as we introduce instrument error and monitor-site location error into our simulations and reduce the number of monitors on which averages are based, attenuation in the health effect estimate is observed. This is more pronounced for log_e_(NO_2_), particularly rural log_e_(NO_2_) than for ozone. This suggests, in line with the findings of others, that attenuation of the relative risk depends not only on instrument error but on the number and placement of monitors [6,16,23] and on the level of spatial variation [6,23,24]. As suggested by Goldman et al. [16], it may be the combination of these sources which determine the ultimate effect on relative risk estimates.

The combined effects of different error sources may also help to explain why contrary to expectation we found no evidence in Tables 2 and 3 (i.e. additive measurement error) of any reduction in statistical power from the use of regional average monitor data based on 2, 3, 5 or 10 monitors per region, with any loss of power most noticeable for the 1 monitor scenario in particular in relation to urban log_e_(NO_2_).

The use of simulated model data produced attenuation in the health effect estimate, which for rural log_e_(NO_2_) was similar to that associated with the scenario of a single regional monitor. However for urban and rural ozone and particularly urban log_e_(NO_2_) regression coefficients were more biased towards the null than for the single monitor case. According to Sheppard et al. [25] classical error can result not only in an attenuated health effect estimate but also lead to a downward bias in the estimation of standard errors and thus to inaccuracy in the coverage of 95% confidence intervals. The appreciable bias in health effect estimates and coverage intervals based on simulated model data for log_e_(NO_2_) therefore implies the presence of predominantly classical rather than Berkson-like error in EMEP-WRF CTM estimates of this pollution metric. In order to investigate this further we attempted using our comparison dataset to decompose random measurement error into its classical-like and Berkson-like components (Additional file 3). Our results suggested that indeed classical error predominates overwhelmingly in the log_e_(NO_2_) CTM data.

The use of NO_2_ rather than log_e_(NO_2_) (i.e. proportional rather than additive measurement error) appeared to lead to a marked improvement in the previously poor coverage probabilities of the model data but further attenuation in health effect estimates based on regional averages. However these regional averages still tended to outperform model data with the possible exception of the 1 monitor per 25 km × 25 km grid square scenario for rural NO_2_ where monitor and model findings were comparable. Unlike additive measurement error whose biasing effect on grid means is effectively adjusted for by including grid as a fixed effect in our time-series analyses, this is not the case when measurement error is proportional. For model data with proportional error therefore it is important to note that our findings may depend to some extent on grid-specific mean pollution levels and the validity of the assumptions we make in simulating them (see Equation 1.2a).

One of the strengths of our simulation approach is that it allows the correlation between time-series in different grids to vary according to the distance between those grids. However, in so doing we make the assumption that spatial dependence is characterised by a single linear function. In our regression analysis of the association between correlation and distance (Figure 1) the addition of a quadratic term was statistically significant for urban and rural ozone and for urban log_e_(NO_2_), although for all three pollutants the incorporation of this non-linearity had a relatively small impact on the percentage of variance explained (explaining an additional 0.2, 1.6 and 1.6 percentage points respectively). We also assume that spatial dependence is independent of direction (i.e. isotropic) and geography (other than a distinction between urban and rural) and does not vary over time. This may not be the case if the study area contains point sources, the outflow from which may vary in direction, with direction varying itself over time due to changing weather conditions. Nevertheless this is an assumption employed by other authors [5,23] in this field, possibly due to the fact that data sufficient to incorporate such features into simulation studies is not readily available or generalizable.

Our simulations allow mean pollution concentrations to vary between grids although we assume that they vary at random and do not take account of the fact that mean pollution concentrations in nearest neighbour grids may be more similar than those at a distance. This could have implications for our results involving proportional measurement error. However, when for each pair of monitoring stations in our observed monitor data set we plotted the absolute difference in site means against distance there was no evidence of a linear relationship whether for log_e_(NO_2_) or ozone, urban or rural. Though in some ways reassuring, these findings may nevertheless be insensitive to differences in grid-mean pollution concentrations within urban areas, where for example background levels of NO_2_ tend to increase as one approaches the urban core [26], whilst background levels of O_3_ tend to decrease.

A further limitation is that we use the same variance to generate each within-grid time-series and that time-series, both modelled and monitored, are simulated without seasonal pattern or trend. Hence we do not consider the influence of time-dependent confounding variables nor other confounders or pollutants. However the effects of measurement error in multi-pollutant models [4,27] and in the presence of confounders have been considered by others [25,28].

Although quantitatively the simulation parameters we used (and hence our results) only apply to the EMEP-WRF model v3.7 for the British Isles, the simulation approach is generalizable and may be used in the evaluation of other chemistry-transport models in other areas.

Eulerian CTMs similar to the EMEP model discretize the real world using a fixed horizontal and vertical grid with no explicit information of within-grid variability of emissions. Linear emissions such as roads and/or point sources are averaged to the CTM horizontal resolution. This approximation may limit the model ability to resolve the near sources chemistry and transport which is likely to occur near urban monitor sites. Moreover, the EMEP model was not designed to replicate the complex urban environment. Local dispersion models which can represent the fine-scale complexity of an urban environment are currently available (ADMS, ERG models), however they are very computationally expensive and are limited to specific areas and rely on CTMs for boundary condition in order to capture the regional import/export of pollutants.

The benefit of full temporal and UK coverage and the self-consistency of predicted chemicals parameters should not be underestimated, and perhaps this benefit overcomes the shortage of properly representing the surface urban chemistry.

Our present findings suggest that there may be an appreciable penalty of using CTM data in spatially-resolved epidemiological time-series studies, which for some pollutants in part weighs against the substantial benefits of such modelled data. These advantages include the opportunity to investigate pollutants (e.g. different particle measures) with sparse or zero monitor coverage, or pollutants from specific sources with direct relevance to policy formulation and evaluation, or the potential consequences from alternative future scenarios. For the simulations incorporating additive measurement error (Tables 2 and 3) and the input data used in this work, we found that monitor data out-performed model data in urban areas and in areas with at least 2 monitors per 25 km × 25 km grid-square but that the performance of monitor and model data for log_e_(NO_2_), at least in terms of power and attenuation in the regression coefficient, was similar in rural areas with only 1 monitor per 25 km × 25 km grid-square. However, it is important to be clear that the impact of ‘measurement’ error as assessed in this paper is only one aspect of data performance relevant to the use of modelled versus monitored data in epidemiological studies, and that monitored data themselves, typically characterised by sparse data from preferential (similar type) locations with measurement errors and often missing values, also have their limitations which are often ignored. High resolution CTMs are continually being developed and our study suggests that further assessment of model error impact - which includes statistical simulation – as well as improved understanding of the performance of monitored data, would be useful.

## Conclusions

Even if correlations between model and monitor data appear reasonably strong, additive classical measurement error in model data may lead to appreciable bias in health effect estimates. As process-based air pollution models become more widely used in epidemiological time-series analysis because of their advantages in terms of geographical coverage and their potential to provide complete time-series for all pollutant species of interest, assessments of error impact which include statistical simulation may be useful.

## Abbreviations

CTM: Chemistry-transport model.

## Competing interests

MRH, RMD and MV have an academic interest in the EMEP-WRF CTM and its development. There are no other conflicts of interest.

## Authors’ contributions

BKB contributed to the design of the study, analysed the data, carried out the simulations and took the lead in drafting the paper. BA provided theoretical statistical expertise and contributed to the design and concept of the study. RWA and PW contributed to the design and concept of the study. MRH and RMD assembled the model data and the model-monitor comparison data sets. MV is the main developer of the EMEP-WRF regional chemistry-transport model and produced the model output. All authors contributed to the drafting of the paper, the interpretation of results and read and approved the final manuscript.

## Pre-publication history

The pre-publication history for this paper can be accessed here:

http://www.biomedcentral.com/1471-2288/13/136/prepub

## Supplementary Material

Additional file 1 Estimating instrument/location error (example urban ozone). Click here for file

Additional file 2 Predicting bias in the health effect estimate from theory. Click here for file

Additional file 3 Investigating the magnitude and components of measurement error. Click here for file
