# Supplementary material for: Measurement error in time-series analysis: a simulation study comparing modelled and monitored data
Source: BMC Med Res Methodol. 2013 Nov 13;13:136. doi: 10.1186/1471-2288-13-136 (PMC3871053; doi:10.1186/1471-2288-13-136)
Supplement: Additional file 1 — Estimating instrument/location error (example urban ozone). [file 1471-2288-13-136-S1.docx]

**Additional file 1 - Estimating instrument / location error (example urban ozone)**

From the equation in Figure 1(a), the constant term is an estimate of the Pearson correlation $\left( \rho\right)$between repeat observations at the same monitor i.e. instrument error. If we assume that within a 5 km by 5 km grid, we have two time-series $X_{a}$ and $X_{b}$ measured with classical error and that each time-series is an approximation of the “true” time-series $X^{*}$ for that grid measured without error, then

$$X_{a}= X^{*}+ E_{a}$$

$$X_{b}= X^{*}+ E_{b}$$

Where both $E_{a}$ and $E_{b}$ are independent $\varepsilon_{a,t}$ $\sim N\left( 0,\sigma_{\mathrm{err}}^{2} \right)$ , $\varepsilon_{b,t}$ $\sim N\left( 0,\sigma_{\mathrm{err}}^{2} \right)$ and ${x_{t}}^{*}\sim N\left( \mu,\sigma_{w}^{2} \right).$ Goldman et al. [5] noted that:

$E\left[ \rho_{X_{a},X^{*}} \right]=$ $E\left[ \rho_{X_{b},X^{*}} \right]=\sqrt{\left( E\left[ \rho_{X_{a},X_{b}} \right] \right)}=\sqrt{\frac{\sigma_{w}^{2}}{\sigma_{err}^{2}+ \sigma_{w}^{2}}}$ (1.3)

**Assuming only instrument error**

Using 1.3 and noting from Figure (1a) that $E\left[ \rho_{X_{a},X_{b}} \right]=0.93031$

$$\sqrt{\left( E\left[ \rho_{X_{a},X_{b}} \right] \right)}=\sqrt{\frac{\sigma_{w}^{2}}{\sigma_{err}^{2}+ \sigma_{w}^{2}}}=\sqrt{0.93031}=0.96453$$

If the within-grid standard deviation of the measured time-series is $25.27772$, the standard deviation of the “true” time series is $25.27772\times0.96453=24.38112$ and $\sigma_{err}= 6.67264$

**Assuming both instrument error and monitor-site location error within grid**

Using 1.3 and noting that $E[P]=0.93031-0.00080\times D$ and $D=2.6$ (estimated average distance between any two points within a 5 km by 5 km grid)

$$\sqrt{\left( E\left[ \rho_{X_{a},X_{b}} \right] \right)}=\sqrt{\frac{\sigma_{w}^{2}}{\sigma_{err}^{2}+ \sigma_{w}^{2}}}=\sqrt{0.93031-0.00080\times2.6}=0.96345$$

If the within-grid standard deviation of the measured time-series is $25.27772$, the standard deviation of the “true” time-series is $25.27772\times0.96345=24.35382$ and $\sigma_{err}=6.77160$
