# Supplementary material for: Measurement error in time-series analysis: a simulation study comparing modelled and monitored data
Source: BMC Med Res Methodol. 2013 Nov 13;13:136. doi: 10.1186/1471-2288-13-136 (PMC3871053; doi:10.1186/1471-2288-13-136)
Supplement: Additional file 2 — Predicting bias in the health effect estimate from theory. [file 1471-2288-13-136-S2.docx]

**Additional file 2 – Predicting bias in the health effect estimate from theory**

Let us suppose that we have two time-series represented by the vectors $X$ and $V.$ We assume $X$ is a time-series of monitor data measured with classical error within a specific 5 km by 5 km grid-square and that it approximates the “true” time-series $X^{*}$ in that grid-square such that:

$X= X^{*}+ E$ and $\varepsilon_{t}\sim N\left( 0,\sigma_{err}^{2} \right)$ (1.4)

With respect to $V$ we assume only that $E$ is independent of both $X^{*}$ and $V.$

i.e. $cov\left( E, V \right)=0$ and $cov\left( X, V \right)=cov\left( X^{*}, V \right)$ (1.5)

Thus $V$ could be a time-series of model data within the same grid square as $X$ or a time-series of monitor data in a different grid-square to $X.$

Given 1.4 and Goldman et al. [5]

$$\left( E\left[ \rho_{XX^{*}} \right] \right)^{2}= \frac{var(X^{*})}{var(X)}$$

Given 1.4 and 1.5

$$\left( E\left[ \rho_{VX^{*}} \right] \right)^{2}=\frac{{cov(V,X^{*})}^{2}}{var\left( V \right)var(X^{*})}= \frac{{cov(V,X)}^{2}}{var\left( V \right)var(X^{*})}$$

Therefore

$$\left( E\left[ \rho_{XX^{*}} \right] \right)^{2}\times\left( E\left[ \rho_{VX^{*}} \right] \right)^{2}= \left\{ \frac{{cov\left( V,X \right)}^{2}}{var\left( V \right)var\left( X^{*} \right)} \right\}\times\frac{var\left( X^{*} \right)}{var\left( X \right)}$$

$$= \frac{{cov(V,X)}^{2}}{var\left( V \right)var(X)}= \left( E\left[ \rho_{VX} \right] \right)^{2}$$

$i.e. \left( E\left[ \rho_{VX^{*}} \right] \right)^{2}=\left( E\left[ \rho_{VX} \right] \right)^{2}/\left( E\left[ \rho_{XX^{*}} \right] \right)^{2}$ (1.6)

The regression calibration formula [3,5], for estimating attenuation in the regression coefficient due to measurement error which may be Berkson, classical, or a combination, can be expressed as:

$\beta_{V}=\beta^{*}\times\frac{cov\left( V,X^{*} \right)}{var\left( V \right)}=\beta^{*}\times\frac{cov\left( V,X \right)}{var\left( V \right)}$ (1.7)

Or, equivalently,

$\beta_{V}=\beta^{*}\times\frac{cov\left( V,X^{*} \right)}{var\left( V \right)}= \beta^{*}\times\left\{ \frac{\rho_{VX^{*}}}{sd\left( V \right)}\times sd(X^{*}) \right\}$ (1.8)

**Predicting bias for the 1-monitor simulation scenario**

The average distance between any two points in a 25 km by 25 km grid-square is estimated by simulation to be approximately 13.04 km. Thus if $V$ is a time-series of pollution data from a single monitor within a 25 km by 25 km square and $V$ is used as a surrogate for each of the constituent 5 km by 5 km grid-squares the average $\rho_{VX}$ across the 25 grid-squares can be estimated by substituting $D=13.04$ in the appropriate equation in Figure 1 and then the average $\rho_{VX^{*}}$ can be estimated using (1.6).
