# Supplementary material for: Measurement error in time-series analysis: a simulation study comparing modelled and monitored data
Source: BMC Med Res Methodol. 2013 Nov 13;13:136. doi: 10.1186/1471-2288-13-136 (PMC3871053; doi:10.1186/1471-2288-13-136)
Supplement: Additional file 3 — Investigating the magnitude and components of measurement error. [file 1471-2288-13-136-S3.docx]

**Additional file 3 – Investigating the magnitude and components of measurement error**

In order to aid our interpretation of the CTM simulation results we used our observed monitor and CTM data and methods/notation outlined in Additional file 2 in order to obtain estimates of $var\left( V-X^{*} \right)$ and also a classical-like component (CC) and a Berkson-like component (BC) of this error variance. Our argument for these estimates, which are subsidiary to the simulations carried out in the main paper, is heuristic:

Given that for pure classical random error

$cov\left( V-X^{*}, V \right)>0 and cov\left( V-X^{*},X^{*} \right)=0$

and that for pure classical Berkson random error

$cov\left( V-X^{*}, X^{*} \right)<0 and cov\left( V-X^{*},V \right)=0$

We decompose $var\left( V-X^{*} \right)$ as follows:

$var\left( V-X^{*} \right)=cov\left( V-X^{*},V \right)+\{-cov\left( V-X^{*},X^{*} \right)\}$ = CC + BC

where

CC=$cov\left( V-X^{*}, V \right)=var\left( V \right)-cov(V,X^{*})$

BC=$\left\{ -cov\left( V-X^{*},X^{*} \right) \right\}=var\left( X^{*} \right)-cov(V,X^{*})$

Given that $cov\left( V,X^{*} \right)=cov\left( V,X \right)$ (Additional file 2) and $var\left( X^{*} \right)=var\left( X \right)-var(E)$ (Additional files 1 and 2), all the quantities in these expressions for CC and BC are estimable from the observed data. Where these expressions gave negative values we set the estimate to zero.

The resulting estimates are given in the table below.

| Variables | Site type | Source of data | Classical-like standard deviation  $\sqrt{CC}$ | Berkson-like standard deviation  $\sqrt{BC}$ | Total error  standard deviation  $\sqrt{var\left( V-X^{*} \right)}$ |
| --- | --- | --- | --- | --- | --- |
| Daily maximum running 8-hour mean O_3_ | Rural | 25 Monitors per region | 8.429 | NA^¶^ | 8.429 |
|  |  | CTM | 9.230 | 7.926 | 12.166 |
|  | Urban | 25 Monitors per region | 6.772 | NA^¶^ | 6.772 |
|  |  | CTM | 9.608 | 11.718 | 15.154 |
| log_e_(Daily maximum  1-hour NO_2_) | Rural | 25 Monitors per region | 0.437 | NA^¶^ | 0.437 |
|  |  | CTM | 0.573 | 0.0 | 0.461 |
|  | Urban | 25 Monitors  per region | 0.256 | NA^¶^ | 0.256 |
|  |  | CTM | 0.470 | 0.0 | 0.430 |

¶ For monitor data (1 monitor per 5 km x 5km grid-square) error is all classical by assumption and $CC=\sigma_{err}^{2}$ (see Additional file 1)

- The error in CTM estimates appears about equally Berkson and classical for ozone, and wholly classical for log_e_(NO_2_).
- Negative estimates of BC were obtained for both urban and rural CTM log_e_(NO_2_). These suggest that some of the measurement error in CTM log_e_(NO_2_) data may be non-random.
